# Penalized regression with multiple sources of prior effects

**DOI:** 10.1093/bioinformatics/btad680

**Published:** 2023-11-10

**Authors:** Armin Rauschenberger, Zied Landoulsi, Mark A van de Wiel, Enrico Glaab

**Affiliations:** Luxembourg Centre for Systems Biomedicine (LCSB), University of Luxembourg, 4362 Esch-sur-Alzette, Luxembourg; Luxembourg Centre for Systems Biomedicine (LCSB), University of Luxembourg, 4362 Esch-sur-Alzette, Luxembourg; Department of Epidemiology and Data Science (EDS), Amsterdam University Medical Centers (Amsterdam UMC), 1081 HV Amsterdam, The Netherlands; Luxembourg Centre for Systems Biomedicine (LCSB), University of Luxembourg, 4362 Esch-sur-Alzette, Luxembourg

## Abstract

**Motivation:**

In many high-dimensional prediction or classification tasks, complementary data on the features are available, e.g. prior biological knowledge on (epi)genetic markers. Here we consider tasks with numerical prior information that provide an insight into the importance (weight) and the direction (sign) of the feature effects, e.g. regression coefficients from previous studies.

**Results:**

We propose an approach for integrating multiple sources of such prior information into penalized regression. If suitable co-data are available, this improves the predictive performance, as shown by simulation and application.

**Availability and implementation:**

The proposed method is implemented in the R package transreg (https://github.com/lcsb-bds/transreg, https://cran.r-project.org/package=transreg).

## 1 Background

For many biomedical prediction or classification studies, there is a previous study with a similar target and a similar high-dimensional feature space, e.g. hundreds of microRNAs (miRNAs), thousands of genes, or millions of single-nucleotide polymorphisms (SNPs). Given a trained model from a previous study, we could use it to obtain predicted values or predicted probabilities for the study of interest, but these predictions are only reliable if the two studies have the same target, the same features, and the same population. However, we expect the feature-target effects from two studies to be strongly correlated in more situations: slightly different targets (e.g. disease status versus disease stage), slightly different features (e.g. imperfectly overlapping feature space, different measurement technique), slightly different populations (e.g. hospitalized versus non-hospitalized patients), or even different modelling approaches (e.g. simple regression versus multiple regression). As it is challenging to estimate feature-target effects in high-dimensional settings, it might be advantageous to use results from previous studies as prior information for the study of interest.

Consider two prediction or classification problems, each one with a target vector and a feature matrix (samples in the rows, features in the columns). Suppose that both feature matrices cover the same features (each column in the first matrix corresponds to a column in the second matrix). In two special cases, the two problems reduce to a single problem: (i) if both problems have the same target and concern samples from the same population, they are in essence one ‘single-target’ problem (combine target vectors and feature matrices by rows, respectively), potentially with batch effects; and (ii) if both problems concern the same samples, they are in essence one ‘multi-target’ problem (combine target vectors by columns, feature matrices are the same). In other cases, however, we might be in a transfer learning setting (Table 1).

**Table 1.** Abstract representation of the dataset of interest (without asterisk, black) and an additional dataset (with asterisk, grey).^a^

$$\begin{array}{ccc} \text{Single-target}\;\text{learning} & \text{Multi-target}\;\text{learning} & \text{Transfer}\;\text{learning}\\ \begin{array}{ccc} \left(\begin{array}{c} y_{1}\\ \vdots \\ y_{n}\\ y_{1}^{*}\\ \vdots \\ y_{m}^{*} \end{array}\right) & ⇐ & \left(\begin{array}{ccc} x_{11} & \cdots & x_{1p}\\ \vdots & & \vdots \\ x_{n1} & \cdots & x_{np}\\ x_{11}^{*} & \cdots & x_{1p}^{*}\\ \vdots & & \vdots \\ x_{m1}^{*} & \cdots & x_{mp}^{*} \end{array}\right) \end{array} & \begin{array}{ccc} \left(\begin{array}{cc} y_{1} & y_{1}^{*}\\ \vdots & \vdots \\ y_{n} & y_{n}^{*} \end{array}\right) & ⇐ & \left(\begin{array}{ccc} x_{11} & \cdots & x_{1p}\\ \vdots & & \vdots \\ x_{n1} & \cdots & x_{np} \end{array}\right) \end{array} & \begin{array}{ccc} \left(\begin{array}{c} y_{1}\\ \vdots \\ y_{n} \end{array}\right) & ⇐ & \left(\begin{array}{ccc} x_{11} & \cdots & x_{1p}\\ \vdots & & \vdots \\ x_{n1} & \cdots & x_{np} \end{array}\right)\\ & & \;\;⇕\;\;\;\;⇕\;\;\;\;⇕\;\;\\ \left(\begin{array}{c} y_{1}^{*}\\ \vdots \\ y_{m}^{*} \end{array}\right) & ⇐ & \left(\begin{array}{ccc} x_{11}^{*} & \cdots & x_{1p}^{*}\\ \vdots & & \vdots \\ x_{m1}^{*} & \cdots & x_{mp}^{*} \end{array}\right) \end{array} \end{array}$$

a Single-target learning (left): same targets, same features${}^{*}$, different samples (from one population). Multi-target learning (centre): different targets, same features${}^{*}$, same samples. Transfer learning (right): same or different targets, matched features${}^{*}$, different samples (from one or two populations). ${}^{*}$Partially overlapping feature spaces are also possible. See, for example, Rauschenberger and Glaab (2021) for multi-target learning or Section 2.8 “Extension” for transfer learning.

In such settings—two or more regression problems with related targets and matched features—it might be possible to transfer information from one problem to another. Transfer learning, in contrast to multi-target regression and seemingly unrelated regressions, addresses related regression problems not for the same but for different samples. If the regression problems are sufficiently related to each other, we expect their regression coefficients to be correlated (positively or negatively). When fitting the regression model of interest, we could therefore account for the estimated regression coefficients from the other model. Transferring information on the importance and the direction of the feature effects, we could potentially increase the predictive performance.

Jiang *et al.* (2016) proposed the prior lasso to account for prior information in high-dimensional predictive modelling. Their method involves a preprocessing step and a weighting step. In the preprocessing step, the prior information is used to predict the target from the features. They present a solution for one set of prior effects from a closely related study (multiplying the feature matrix by the prior effects), but extensions to multiple sets of prior effects or loosely related studies may be feasible. Let ***y*** represent the target and let ${\hat{y}}_{\text{prior}}$ represent the fitted values based on the prior information. In the weighting step, they minimize the penalized combined likelihood $L(x,y;\beta )+\eta L(x,{\hat{y}}_{\text{prior}};\beta )-\rho (\lambda ;\beta )$ with respect to the coefficients β, where η≥0 (balance) and λ≥0 (regularization). If the balancing hyperparameter *η* is larger than zero, the prior predictions ${\hat{y}}_{\text{prior}}$ influence the estimation of the parameters β.

Dhruba (2021) proposed a transfer learning method based on distribution mapping. Even if features or targets follow different distributions in two datasets, it is possible to build a predictive model using the first dataset and make predictions for the second dataset. Requiring matched features and targets in the source dataset and unmatched features and targets in the target dataset, their method transfers (i) features from the target to the source domain and (ii) predictions from the source to the target domain. In contrast, we consider transfer learning settings with matched features and targets in the target dataset.

Tian and Feng (2022) proposed and implemented transfer learning for ridge and lasso regression. Their transfer learning algorithm involves two steps: (i) estimating common coefficients for the target dataset and the transferable source datasets ($\hat{\omega }$) and (ii) estimating the deviations from the common coefficients to the target coefficients ($\hat{\delta }$). Both steps together lead to the estimated target coefficients ($\hat{\beta }=\hat{\omega }+\hat{\delta }$). Before applying their transfer learning algorithm, Tian and Feng (2022) apply a transferable source detection algorithm to exclude source datasets that are too different from the target dataset. This avoids that non-transferable sources render the common coefficients misleading for the target dataset (‘negative transfer’). In the case of lasso regularization in the two steps, there is sparsity in the common estimates as well as in the deviations from the common estimates to the target estimates (and thereby also in the target estimates).

The method from Tian and Feng (2022) requires not only the target dataset but also the source dataset(s). However, data protection regulations or restrictive data sharing policies might prevent researchers from accessing a source dataset, or the available storage or processing capacity might be insufficient for analysing massive source datasets. Although federated transfer learning (Liu *et al.* 2020) enables the joint analysis of related datasets without sharing them, it is often not practically feasible for biomedical researchers to set up corresponding collaborative analyses with multiple data holders. There is therefore a need for transfer learning methods that require neither direct nor indirect access to the source data but only to the complementary data (co-data) derived from the source data. Such methods allow us to exploit publicly available summary statistics from external studies, e.g. *P*-values and effect sizes from a genome-wide association study (GWAS), to increase the predictive performance in the study of interest.

We propose a two-step transfer learning method, modelling with and without co-data in the first step and combining different models in the second step. Unless the source and target datasets are very similar, the coefficients from the source dataset(s) will not fit well to the target dataset. We therefore propose to calibrate these coefficients—preserving their signs and their order—so that they can be transferred from the source dataset(s) to the target dataset. Additionally, we also estimate the coefficients directly from the target dataset, ignoring the co-data. The calibrated coefficients from the source dataset(s) as well as the estimated coefficients from the target dataset allow us to predict the outcome from the features. Finally, we combine the linear predictors from the models with and without co-data and calculate either predicted values (linear regression) or predicted probabilities (logistic regression).

Kawaguchi *et al.* (2022) proposed a related transfer learning method. This method penalizes (i) the differences between the coefficients and weighted sums of the prior effects (one difference for each feature) and (ii) the weights for these weighted sums (one weight for each set of prior effects), possibly with two different penalties (e.g. ridge and lasso). It not only allows for high-dimensional data (i.e. more features than samples) but also for high-dimensional co-data (i.e. more sets of prior effects than samples). When the unknown feature effects are non-linearly related to an important source of prior effects, however, this method might leave room for improvement.

In a related transfer learning setting, prior information is only available on the importance but not on the direction of the feature effects, i.e. with complementary data consisting of prior weights rather than prior effects. In the generalized linear model framework, the weighted lasso (Bergersen *et al.* 2011), the feature-weighted elastic net (Tay *et al.* 2023), and penalized regression with differential shrinkage (Zeng *et al.* 2021) account for prior weights in the penalty function, through feature-specific penalty factors or feature-specific regularization parameters. Adaptive group-regularized ridge regression (van de Wiel *et al.* 2016) is not only applicable to categorical co-data but also to numerical co-data (prior weights), by the means of creating groups of features from numerical co-data and forcing the group-penalties to be monotonically decreasing. An extension from van Nee *et al.* (2021) makes this approach even more suitable for numerical co-data. For single sources of co-data, it might be possible to extend these methods to prior information on the importance as well as the direction of feature effects by imposing sign constraints on the coefficients. Prior weights have not only been exploited in regression analysis, e.g. co-data moderated random forests (te Beest *et al.* 2017) adapt the sampling probabilities of the features to the prior weights.

## 2 Materials and methods

### 2.1 Model

Suppose one target and *p* features are available for *n* samples, with many more features than samples (p≫n). We index the samples by *i* in {1,…,n} and the features by *j* in {1,…,p}. Our aim is to estimate the generalized linear model:


$$E[y_{i}]=h^{-1}\left(\beta_{0}+\sum_{j=1}^{p}\beta_{j}x_{ij}\right)\;.$$

For any sample *i*, the model expresses the expected value of its target (*y_i_*) as a function of its features ($x_{i1},\ldots ,x_{ip}$). The link function h(·) depends on the family of distributions for the target (Gaussian: identity, binomial: logit, Poisson: log). In the linear predictor, *β*_0_ represents the unknown intercept, and *β_j_* represents the unknown slope of feature *j* (i.e. the effect of the feature on the linear predictor of the target). Given the estimated intercept ${\hat{\beta }}_{0}^{⋆}$ and the estimated slopes $\left\{{\hat{\beta }}_{1}^{⋆},\ldots ,{\hat{\beta }}_{p}^{⋆}\right\}$, we could predict the target of previously unseen samples:


$${\hat{y}}_{i}=h^{-1}\left({\hat{\beta }}_{0}^{⋆}+{\sum_{j=1}^{p}\hat{\beta }}_{j}^{⋆}x_{ij}\right)\;.$$

### 2.2 Co-data

Suppose *m* sources of co-data are available, indexed by *k* in {1,…,m}, with many fewer sources than samples (m≪n). Let *z_jk_* indicate the prior effect from source *k* for feature *j*. Our method is designed for quantitative co-data that provide an insight into the importance (absolute value) and the direction (sign) of the feature effects. Each set of prior effects ($-1\leq z_{{}^{\circ}k}\leq 1$) is assumed to be positively correlated with the true coefficients ($\text{cor}(z_{{}^{\circ}k},\beta )>0$). For any source of co-data, the prior effects may be re-scaled (not re-centred), for example to the interval from −1 to +1. In other words, the proposed method is invariant under multiplication of the prior effects by a positive scalar (z→c×z where *c *>* *0). We explain in the next section why this is important.

It might seem trivial to also allow for co-data that only provide an insight into the importance but not the direction of the feature effects (i.e. prior weights instead of prior effects). Each set of prior weights ($0\leq z_{{}^{\circ}k}\leq 1$) is assumed to be positively correlated with the true absolute coefficients ($\text{cor}(z_{{}^{\circ}k},|\beta |)>0$). To obtain prior effects, one might want to assign the signs of the Spearman correlation coefficients between the target and the features to the prior weights. However, marginal effects and conditional effects can have opposite signs. If we wanted to extend our approach to prior weights, we would have to discover the signs inside the calibration procedure (see below), which would be related to high-dimensional regression with binary coefficients (Gamarnik and Zadik 2017).

### 2.3 Base-learners with co-data

Suppose we are in a transfer learning setting with two prediction or classification problems. For simplicity, we assume that the features do not differ in scale between the two problems. For illustration, we consider two artificial situations (where we would not use transfer learning in practice): (i) if both problems concern the same target on the same scale and the samples come from the same population, we could use the estimated regression coefficients from one problem to make predictions for the other problem; and (ii) if the two problems concern the same target on different scales, we could also recycle the estimated regression coefficients, but we would have to adjust for the different scales.

When transferring estimated regression coefficients from one problem to another problem, it might not only be necessary to change their scale but it might also be beneficial to change their shape. For example, it might be that for one problem weak and strong effects matter, while for the other problem only strong effects matter. We should therefore also be able to make differences between small coefficients more important or less important than those between large coefficients. We propose two calibration methods, namely exponential and isotonic calibration, to adapt the prior information to the data. For each source of co-data *k*, both calibration methods estimate the model:


$$E[y_{i}]=h^{-1}\left(\alpha_{k}+\sum_{j=1}^{p}\gamma_{jk}x_{ij}\right)\;,$$

where the calibrated prior effects $\left\{{\hat{\gamma }}_{1k},\ldots ,{\hat{\gamma }}_{pk}\right\}$ depend on the initial prior effects $\left\{z_{1k},\ldots ,z_{pk}\right\}$. The difference between exponential and isotonic calibration is how the former depend on the latter.

- Exponential calibration: Let $\gamma_{jk}=\theta_{k}\text{sign}(z_{jk})|z_{jk}{|}^{\tau_{k}}$, for *j* in {1,…,p}, where the factor *θ_k_* and the exponent *τ_k_* are non-negative real numbers ($\theta_{k}\geq 0$ and $\tau_{k}\geq 0$). We first fit one simple non-negative regression for different values of *τ_k_* (i.e. estimate *α_k_* and *θ_k_* given *τ_k_*), and then optimize *τ_k_*. Once *θ_k_* and *τ_k_* have been estimated, the initial prior effects *z_jk_* determine the final prior effects ${\hat{\gamma }}_{jk}={\hat{\theta }}_{k}\text{sign}(z_{jk})|z_{jk}{|}^{{\hat{\tau }}_{k}}$, for all *j* in {1,…,p}. The estimated factor ${\hat{\theta }}_{k}$ and the estimated exponent ${\hat{\tau }}_{k}$ allow the model to change the scale and the shape of the prior effects. For example, ${\hat{\theta }}_{k}=0$ sets them to zero, $|{\hat{\theta }}_{k}|<1$ makes them smaller, $|{\hat{\theta }}_{k}|>1$ makes them larger, ${\hat{\tau }}_{k}=0$ sets them to the same value, ${\hat{\tau }}_{k}<1$ makes (absolutely) large ones more similar, and ${\hat{\tau }}_{k}>1$ makes (absolutely) small ones more similar. If one or more sets of prior effects might be negatively associated with the true coefficients, we could remove the non-negativity constraints from the simple regressions (allowing ${\hat{\theta }}_{k}<0$ to invert the signs of the prior effects).
- Isotonic calibration: We estimate $\left\{\gamma_{1k},\ldots ,\gamma_{pk}\right\}$ under the constraint that the signs of the initial prior effects *z_jk_* determine the signs of the final prior effects ${\hat{\gamma }}_{jk}$ (i.e. ${\hat{\gamma }}_{jk}=0|z_{jk}=0$, ${\hat{\gamma }}_{jk}\geq 0|z_{jk}>0,\;{\hat{\gamma }}_{jk}\leq 0|z_{jk}<0$) and under the constraint that the order of the initial prior effects determines the order of the final prior effects (i.e. ${\hat{\gamma }}_{jk}\geq {\hat{\gamma }}_{lk}|z_{jk}\geq z_{lk},\;{\hat{\gamma }}_{jk}\leq {\hat{\gamma }}_{lk}|z_{jk}\leq z_{lk}$), for all *j* and *l* in {1,…,p}. If one or more sets of prior effects might be negatively associated with the true coefficients, we could fit each model with these constraints and the inverted constraints, and then select the better fit.
- To make optimization more efficient, we rewrite the sign- and order-constrained problem as a sign-constrained problem (see Table 2). For each source of co-data, we order the columns of the feature matrix by increasing values of the prior effects. Suppose the first *q* columns correspond to negative prior effects and the last *p–q* columns correspond to non-negative prior effects. We take the cumulative sum of the feature columns from left to right for the former (columns 1 to *q*) and from right to left for the latter (columns *p* to *q *+* *1). We then estimate the coefficients on the left under non-positivity constraints, and those on the right under non-negativity constraints. Formally, the model equals
  $$E[y_{i}]=h^{-1}\left(\alpha_{k}+\sum_{j=1}^{p}\delta_{jk}w_{ij}\right)\;,$$

**Table 2.** Isotonic calibration.^a^

$$\begin{array}{cccccc} \left(i\right) & x_{{}^{\circ},1},z_{1,k} & x_{{}^{\circ},2},z_{2,k} & \cdots & x_{{}^{\circ},q-1},z_{q-1,k} & x_{{}^{\circ},q},z_{q,k}\\ \left(\text{ii}\right) & x_{{}^{\circ},(1)} & x_{{}^{\circ},(2)} & \cdots & x_{{}^{\circ},(q-1)} & x_{{}^{\circ},(q)}\\ \left(\text{iii}\right) & \begin{array}{c} w_{{}^{\circ},1}=\\ x_{{}^{\circ},(1)} \end{array} & \begin{array}{c} w_{{}^{\circ},2}=\\ x_{{}^{\circ},(1)}+x_{{}^{\circ},(2)} \end{array} & \cdots & \begin{array}{c} w_{{}^{\circ},q-1}=\\ x_{{}^{\circ},(1)}+\cdots +x_{{}^{\circ},(q-1)} \end{array} & \begin{array}{c} w_{{}^{\circ},q}=\\ x_{{}^{\circ},(1)}+\cdots +x_{{}^{\circ},(q)} \end{array}\\ {\left(\text{iii}\right)}^{*} & {\hat{\delta }}_{1,k} & {\hat{\delta }}_{2,k} & \cdots & {\hat{\delta }}_{q-1,k} & {\hat{\delta }}_{q,k}\\ {\left(\text{ii}\right)}^{*} & \begin{array}{c} {\hat{\gamma }}_{(1),k}=\\ {\hat{\delta }}_{1,k}+\cdots +{\hat{\delta }}_{q,k} \end{array} & \begin{array}{c} {\hat{\gamma }}_{(2),k}=\\ {\hat{\delta }}_{2,k}+\cdots +{\hat{\delta }}_{q,k} \end{array} & \cdots & \begin{array}{c} {\hat{\gamma }}_{(q-1),k}=\\ {\hat{\delta }}_{q-1,k}+{\hat{\delta }}_{q,k} \end{array} & \begin{array}{c} {\hat{\gamma }}_{(q),k}=\\ {\hat{\delta }}_{q,k} \end{array}\\ {\left(i\right)}^{*} & {\hat{\gamma }}_{1,k} & {\hat{\gamma }}_{2,k} & \cdots & {\hat{\gamma }}_{q-1,k} & {\hat{\gamma }}_{q,k} \end{array}$$

$$\begin{array}{cccccc} \left(i\right) & x_{{}^{\circ},q+1},z_{q+1,k} & x_{{}^{\circ},q+2},z_{q+2,k} & \cdots & x_{{}^{\circ},p-1},z_{p-1,k} & x_{{}^{\circ},p},z_{p,k}\\ \left(\text{ii}\right) & x_{{}^{\circ},(q+1)} & x_{{}^{\circ},(q+2)} & \cdots & x_{{}^{\circ},(p-1)} & x_{{}^{\circ},(p)}\\ \left(\text{iii}\right) & \begin{array}{c} w_{{}^{\circ},q+1}=\\ x_{{}^{\circ},(q+1)}+\cdots +x_{{}^{\circ},(p)} \end{array} & \begin{array}{c} w_{{}^{\circ},q+2}=\\ x_{{}^{\circ},(q+2)}+\cdots +x_{{}^{\circ},(p)} \end{array} & \cdots & \begin{array}{c} w_{{}^{\circ},p-1}=\\ x_{{}^{\circ},(p-1)}+x_{{}^{\circ},(p)} \end{array} & \begin{array}{c} w_{{}^{\circ},p}=\\ x_{{}^{\circ},(p)} \end{array}\\ {\left(\text{iii}\right)}^{*} & {\hat{\delta }}_{q+1,k} & {\hat{\delta }}_{q+2,k} & \cdots & {\hat{\delta }}_{p-1,k} & {\hat{\delta }}_{p,k}\\ {\left(\text{ii}\right)}^{*} & \begin{array}{c} {\hat{\gamma }}_{(q+1),k}=\\ {\hat{\delta }}_{q+1,k} \end{array} & \begin{array}{c} {\hat{\gamma }}_{(q+2),k}=\\ {\hat{\delta }}_{q+1,k}+{\hat{\delta }}_{q+2,k} \end{array} & \cdots & \begin{array}{c} {\hat{\gamma }}_{(p-1),k}=\\ {\hat{\delta }}_{q+1,k}+\cdots +{\hat{\delta }}_{p-1,k} \end{array} & \begin{array}{c} {\hat{\gamma }}_{(p),k}=\\ {\hat{\delta }}_{q+1,k}+\cdots +{\hat{\delta }}_{p,k} \end{array}\\ {\left(i\right)}^{*} & {\hat{\gamma }}_{q+1,k} & {\hat{\gamma }}_{q+2,k} & \cdots & {\hat{\gamma }}_{p-1,k} & {\hat{\gamma }}_{p,k} \end{array}$$

a The aim is to (i) estimate the effects of the features under sign and order constraints determined by *q* negative (top) and *p–q* non-negative (bottom) prior effects, i.e. estimate $\gamma_{1k},\ldots ,\gamma_{pk}$ for $x_{1},\ldots ,x_{p}$ under ${\hat{\gamma }}_{jk}=0|z_{jk}=0$, ${\hat{\gamma }}_{jk}\geq 0|z_{jk}>0,\;{\hat{\gamma }}_{jk}\leq 0|z_{jk}<0,\;{\hat{\gamma }}_{jk}\geq {\hat{\gamma }}_{lk}|z_{jk}\geq z_{lk}$, and ${\hat{\gamma }}_{jk}\leq {\hat{\gamma }}_{lk}|z_{jk}\leq z_{lk}$. This can be solved by (ii) estimating the effects of the features ordered by the co-data under sign and order constraints, i.e. estimate $\gamma_{(1),k},\ldots ,\gamma_{(p),k}$ for $x_{\left(1\right)},\ldots ,x_{\left(p\right)}$ under ${\hat{\gamma }}_{(j),k}\leq 0|j\leq p,\;{\hat{\gamma }}_{(j),k}\geq 0|j>p$, and ${\hat{\gamma }}_{(1),k}\leq \ldots \leq {\hat{\gamma }}_{(p),k}$. This in turn can be solved by (iii) estimating the effects of the combined features under sign constraints, i.e. estimate $\delta_{1},\ldots ,\delta_{p}$ for $w_{1},\ldots ,w_{p}$ under ${\hat{\delta }}_{j}\leq 0|j\leq p$ and ${\hat{\delta }}_{j}\geq 0|j>p$. Our algorithm receives the original features and the prior effects (i), orders the features by the prior effects (ii), combines the features (iii), estimates the effects of the combined features (iii)${}^{*}$, calculates the estimated effects of the ordered features (ii)${}^{*}$, and returns the estimated effects of the original features (i)${}^{*}$. ${}^{*}$A row with an asterisk contains the estimates for the features in the row with the same number but without an asterisk.

where $w_{ij}=\sum_{l=1}^{j}x_{i(l)}$ and $\delta_{jk}\leq 0$ for *j* in {1,…,q}, and $w_{ij}=\sum_{l=j}^{p}x_{i(l)}$ and $\delta_{jk}\geq 0$ for *j* in {q+1,…,p}, with the subscript within brackets indicating the order of the prior effects. The linear predictor of the sign-constrained model, i.e. $\alpha_{k}+\sum_{j=1}^{p}\delta_{jk}w_{ij}$, is equivalent to the linear predictor of the order-constrained model, i.e. $\alpha_{k}+\sum_{j=1}^{p}\gamma_{(j)k}x_{i(j)}$, because


$$\begin{array}{c} \sum_{j=1}^{q}\delta_{jk}w_{ij}=\sum_{j=1}^{q}\delta_{jk}\left(\sum_{l=1}^{j}x_{i(l)}\right)\\ =\sum_{j=1}^{q}\left(\sum_{l=j}^{q}\delta_{lk}\right)x_{i(j)}\\ =\sum_{j=1}^{q}\gamma_{(j)k}x_{i(j)}\;,\\ \sum_{j=q+1}^{p}\delta_{jk}w_{ij}=\sum_{j=q+1}^{p}\delta_{jk}\left(\sum_{l=j}^{p}x_{i(l)}\right)\\ =\sum_{j=q+1}^{p}\left(\sum_{l=q+1}^{j}\delta_{lk}\right)x_{i(j)}\\ =\sum_{j=q+1}^{p}\gamma_{(j)k}x_{i(j)}\;. \end{array}$$

After estimating the coefficients of the sign-constrained model by maximum likelihood, we therefore estimate those of the order-constrained model by ${\hat{\gamma }}_{(j)k}=\sum_{l=j}^{q}{\hat{\delta }}_{lk}$ for *j* in {1,…,q} and ${\hat{\gamma }}_{(j)k}=\sum_{l=q+1}^{j}{\hat{\delta }}_{lk}$ for *j* in {q+1,…,p}.

**Figure 1.**
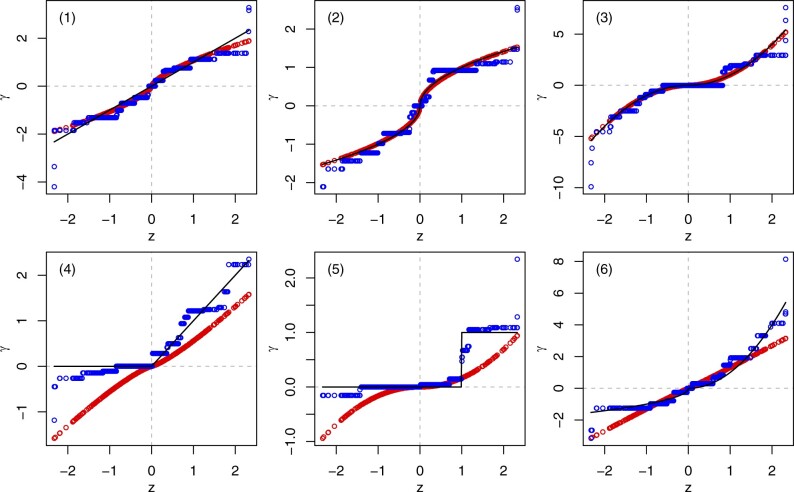
Final prior effects γ (*y*-axis) against initial prior effects ***z*** (*x*-axis), under exponential calibration (red points on continuous curve) and isotonic calibration (blue points on discontinuous curve). The thin black line corresponds to perfect calibration (γ=β). We simulated the feature matrix ***X*** from a standard Gaussian distribution (*n *=* *200, *p *=* *500) and the initial prior effects ***z*** from a trimmed standard Gaussian distribution (trimmed below the 1% and above the 99% quantile). We set the true coefficients to (1) β=z, (2)$\;\beta =\text{sign}(z)\sqrt{\left|z\right|}$, (3)$\;\beta =\text{sign}(z)z^{2}$, (4) β=I[z>0]z, (5) β=I[z>1], or (6)$\;\beta =-I[z\leq 0]\sqrt{\left|z\right|}+I[z>0]z^{2}$. And we simulated the response vector ***y*** from Gaussian distributions with the means η and the variance Var(η), where η=Xβ. While exponential calibration performs slightly better in the first three scenarios (top), isotonic calibration performs much better in the last three scenarios (bottom).

While exponential calibration involves three unknown parameters, namely the intercept *α_k_*, the factor *θ_k_*, and the exponent *τ_k_*, isotonic calibration involves 1+p unknown parameters, namely the intercept *α_k_* and the slopes $\gamma_{k}=\{\gamma_{1k},\ldots ,\gamma_{pk}\}$, for each set of co-data. Figure 1 shows the difference between exponential and isotonic calibration in several empirically assessed scenarios.

After calibration, we pre-assess the utility of each set of co-data. To do this, we calculate the residuals (depending on the family of distributions) between the fitted and the observed targets. We suggest to retain a set of co-data only if the residuals are significantly smaller than those of the intercept-only model (one-sided Wilcoxon signed-rank test) at the nominal 5% level (*P*-value ≤.05).

### 2.4 Base-learners without co-data

We also fit the model without any co-data. We estimate the coefficients by maximizing the penalized likelihood:


$$\hat{\beta }=\underset{\beta }{\text{argmax}}\left\{L(x;\beta )-\rho (\lambda ;\beta )\right\}\;,$$

where L(x,β) is the likelihood and ρ(λ;β) is the penalty. The likelihood depends on the family of distributions (Gaussian, binomial, Poisson), and the penalty can be the ridge (*L*_2_) or the lasso (*L*_1_) penalty. The penalty shrinks the squared (ridge) or absolute (lasso) slopes $\left\{\beta_{1},\ldots ,\beta_{p}\right\}$ towards zero (without penalizing the intercept *β*_0_). We denote the estimated intercept by ${\hat{\beta }}_{0}$ and the estimated slopes by $\left\{{\hat{\beta }}_{1},\ldots ,{\hat{\beta }}_{p}\right\}$.

### 2.5 Cross-validation

We split the samples into 10 folds to perform 10-fold internal cross-validation. In each iteration, we fit the models to nine included folds and predict the target for the excluded fold.

Let the *n *×* m* matrix ${\hat{H}}^{\left(0,\text{cv}\right)}$ represent the feature-dependent part of the cross-validated linear predictors from the models with co-data. Specifically, the entry in row *i* (sample) and column *k* (source of co-data) equals


$$\eta_{ik}^{\left(0,\text{cv}\right)}=0\times {\hat{\alpha }}_{k}^{-\kappa (i)}+\sum_{j=1}^{p}{\hat{\gamma }}_{jk}^{-\kappa (i)}x_{ij}\;,$$

where the superscript −κ(i) indicates that the (ignored) intercept *α_k_* and the slopes *γ_jk_* for *j* in {1,…,p} are estimated without using the fold of sample *i*, as in Rauschenberger and Glaab (2021).

The models without any co-data do not only have 1+p unknown parameters, namely the intercept *β*_0_ and the slopes $\left\{\beta_{1},\ldots ,\beta_{p}\right\}$, but also the unknown hyperparameter *λ*. In each iteration, we fit this model for a decreasing sequence of 100 values for the regularization parameter *λ*, indexed by *l* in {1,…,100}, using the computationally efficient approach from Friedman *et al.* (2010, glmnet).

Accordingly, let the n×100 matrix ${\hat{H}}^{\left(1,\text{cv}\right)}$ represent the cross-validated linear predictors from the model without co-data. Specifically, the entry in row *i* (sample) and column *l* (regularization parameter) equals


$$\eta_{il}^{\left(1,\text{cv}\right)}={\hat{\beta }}_{0}^{-\kappa (i),l}+{\sum_{j=1}^{p}\hat{\beta }}_{j}^{-\kappa (i),l}x_{ij}\;,$$

where the superscripts −κ(i) and *l* indicate that the intercept *β*_0_ and the slopes $\left\{\beta_{1},\ldots ,\beta_{p}\right\}$ are estimated without using the fold of sample *i* and given the regularization parameter *λ_l_*.

To optimize the predictive performance of the co-data independent model, we would select the *λ* that minimizes the cross-validated loss ($\lambda_{\text{min}}$). As we base our predictions not only on the co-data independent model but also on the co-data dependent model(s), $\lambda_{\text{min}}$ might be too small. The reason is that the co-data might be informative to the extent that the co-data independent model requires more penalization. We could let the meta-learner select the optimal *λ* from the whole sequence, but this might render the inclusion and exclusion of co-data dependent models unstable. Our *ad hoc* solution is to include the optimal regularization parameter for the co-data independent model ($\lambda_{\text{min}}$) and a slightly larger one ($\lambda_{1\text{se}}$). The latter is given by the one-standard-error rule, which increases *λ* until the cross-validated loss equals its minimum plus one standard error.

We concatenate ${\hat{H}}^{\left(0,\text{cv}\right)}$ with the columns of ${\hat{H}}^{\left(1,\text{cv}\right)}$ that correspond to $\lambda_{\text{min}}$ and $\lambda_{1\text{se}}$ to obtain the n×(m+2) matrix ${\hat{H}}^{\left(\text{cv}\right)}$. The first *m* columns correspond to the models with co-data, and the last two columns correspond to the model without co-data.

### 2.6 Meta-learner

We combine the base-learners with and without co-data by stacked generalization (Wolpert 1992), on the level of the linear predictors (Rauschenberger *et al.* 2021). In the meta-layer, we regress the target on the cross-validated linear predictors from the base-layer:


$$E[y_{i}]=h^{-1}\left(\omega_{0}+\sum_{k=1}^{m+2}\omega_{k}{\hat{H}}_{ik}^{\left(\text{cv}\right)}\right)\;.$$

Leaving the intercept unrestricted $\left(-\infty <\omega_{0}<+\infty \right)$ but imposing the lower bound zero on the slopes $\left(\omega_{1}\geq 0,\ldots ,\omega_{m+2}\geq 0\right)$, we estimate these coefficients under lasso regularization. Due to the feature selection property of the lasso, a source of co-data can be excluded (${\hat{\omega }}_{k}=0$) or included (${\hat{\omega }}_{k}>0$), where *k* is in {1,…,m}. Similarly, the models without co-data can be excluded (${\hat{\omega }}_{k}=0$) or included (${\hat{\omega }}_{k}>0$), where k=m+1 for the model with $\lambda_{\text{min}}$ and k=m+2 for the model with $\lambda_{1\text{se}}$. The estimated slopes function as weights for the co-data dependent models (${\hat{\omega }}_{1},\ldots ,{\hat{\omega }}_{m}$) and for the co-data independent models (${\hat{\omega }}_{m+1},{\hat{\omega }}_{m+2}$). Thus, we do not only select sources but also weight them according to their relevance.

### 2.7 Interpretation

The coefficients ${\hat{\beta }}_{\text{min}}$ and ${\hat{\beta }}_{1\text{se}}$ give insight into the feature-target effects estimated without co-data, with ${\hat{\beta }}_{\text{min},j}$ and ${\hat{\beta }}_{1\text{se},j}$ representing the effect of feature *j*, where *j* is in {1,…,p}. The coefficients $\hat{\omega }$ give insight into the importance of the sources of co-data, with ${\hat{\omega }}_{k}$ representing the importance of source *k*, where *k* is in {1,…,m}. For a previously unseen sample *i*, the predicted value is:


(1) $$\begin{array}{c} {\hat{y}}_{i}=h^{-1}\left({\hat{\omega }}_{0}+\sum_{k=1}^{m+2}{\hat{\omega }}_{k}{\hat{H}}_{ik}\right)=h^{-1}\left({\hat{\beta }}_{0}^{⋆}+{\sum_{j=1}^{p}\hat{\beta }}_{j}^{⋆}x_{ij}\right)\;,\\ \text{where}\;{\hat{\beta }}_{0}^{⋆}={\hat{\omega }}_{0}+{\hat{\omega }}_{m+1}{\hat{\beta }}_{\text{min},0}+{\hat{\omega }}_{m+2}{\hat{\beta }}_{1\text{se},0}\\ \text{and}\;{\hat{\beta }}_{j}^{⋆}=\left(\sum_{k=1}^{m}{\hat{\omega }}_{k}{\hat{\gamma }}_{jk}\right)+{\hat{\omega }}_{m+1}{\hat{\beta }}_{\text{min},j}+{\hat{\omega }}_{m+2}{\hat{\beta }}_{1\text{se},j}\;. \end{array}$$

Thus, the estimated effect for a feature (${\hat{\beta }}_{j}^{⋆}$) is a weighted sum of estimated coefficients with co-data (${\hat{\gamma }}_{j1},\ldots ,{\hat{\gamma }}_{jm}$) and the estimated coefficients without co-data (${\hat{\beta }}_{\text{min},j},{\hat{\beta }}_{1\text{se},j}$).

Sparse models (few non-zero coefficients) are often considered to be more interpretable than dense models (many non-zero coefficients). While the original coefficients are dense ($\sum_{j=1}^{p}I[{\hat{\beta }}_{j}\neq 0]=p$) or sparse ($\sum_{j=1}^{p}I[{\hat{\beta }}_{j}\neq 0]\ll p$) depending on the choice between ridge and lasso regularization, the weights may contain some zeros due to significance filtering or lasso regularization ($\sum_{k=1}^{m+2}I[{\hat{\omega }}_{k}\neq 0]\leq m+2$). As soon as one set of dense prior effects is selected, however, the combined coefficients also become dense ($\sum_{j=1}^{p}I[{\hat{\beta }}_{j}^{⋆}\neq 0]≲p$). This means that the feature selection property of the lasso is not maintained. We should therefore choose between ridge and lasso regularization (i) to make the model without co-data more predictive or interpretable (ii) or to make the model with co-data more predictive (iii) but not to make the model with co-data more interpretable.

### 2.8 Extension

In some applications, prior information might be available and reliable for some features but missing or unreliable for other features. Note that a missing prior effect can be interpreted as an unreliable prior effect of zero. Although the base-learners with co-data might still be predictive, the meta-learner (weighted average of the base-learners with and without co-data) might be not more predictive than the base-learner without co-data. The reason is that the meta-learner assigns the same weight to all prior effects, rather than more weight to available and reliable prior effects and less weight to missing or unreliable prior effects. We therefore propose an alternative approach for applications with partially informative sources of co-data.

In the following, we use the term ‘meta-features’ for the cross-validated linear predictors from the base learners with co-data. Each meta-feature—one column of the *n *×* m* matrix ${\hat{H}}^{\left(0,\text{cv}\right)}$—corresponds to one source of co-data. In the meta-layer, we regress the target on the meta-features and the base-features:


$$E[y_{i}]=h^{-1}\left(\beta_{0}+\sum_{k=1}^{m}\omega_{k}{\hat{H}}_{ik}^{\left(0,\text{cv}\right)}+\sum_{j=1}^{p}\beta_{j}x_{ij}\right)\;,$$

with non-negativity constraints for the weights for the meta-features $\left(\omega_{1}\geq 0,\ldots ,\omega_{m}\geq 0\right)$ but without constraints for the intercept (*β*_0_) and the slopes for the base-features $\left(\beta_{1},\ldots ,\beta_{p}\right)$.

We estimate the weights for the meta-features and the slopes for the base-features using penalized maximum likelihood:


$$\{\hat{\omega },\hat{\beta }\}=\underset{\left\{\omega ,\beta \right\}}{\text{argmax}}\left\{L(x;\omega ,\beta )-\rho (\lambda ;\beta )\right\}\;,$$

where L(x;ω,β) is the likelihood and ρ(λ;β) is the penalty. We do not penalize the weights for the meta-features (m≪n) but only the slopes for the base-features (p≫n). The more sources of co-data are available, the more it becomes necessary to penalize their weights. But then the weights ω and the slopes β might need differential penalization, for example a lasso penalty for the meta-features (selection of sources) and a ridge penalty for the base-features (many small effects). To make this computationally efficient, we would need a fast cross-validation procedure for multiple penalties (cf. van de Wiel *et al.* 2021) with non-negativity constraints (meta-features) and mixed lasso and ridge penalization (meta-features versus base-features). This extension is therefore only applicable in settings with few sources of co-data.

The predicted value for a previously unseen sample *i* is


(2) $$\begin{array}{c} {\hat{y}}_{i}=h^{-1}\left({\hat{\beta }}_{0}+\sum_{k=1}^{m}{\hat{\omega }}_{k}{\hat{H}}_{ik}^{\left(0\right)}+\sum_{j=1}^{p}{\hat{\beta }}_{j}x_{ij}\right)\\ =h^{-1}\left({\hat{\beta }}_{0}^{⋆}+{\sum_{j=1}^{p}\hat{\beta }}_{j}^{⋆}x_{ij}\right)\;,\\ \text{where}\;{\hat{\beta }}_{0}^{⋆}={\hat{\beta }}_{0}\\ \text{and}\;{\hat{\beta }}_{j}^{⋆}=\left(\sum_{k=1}^{m}{\hat{\omega }}_{k}{\hat{\gamma }}_{jk}\right)+{\hat{\beta }}_{j}\;. \end{array}$$

As the coefficients β are shrunk towards zero but the coefficients ω are not penalized, the combined coefficients $\beta^{⋆}$ are shrunk towards a weighted sum of the calibrated prior effects ($\hat{\gamma }\hat{\omega }$). When the regularization parameter tends to infinity (λ→∞), the estimated deviations from this weighted sum approach zero $\left(\hat{\beta }\to 0\right)$ and the combined estimates approach this weighted sum $\left({\hat{\beta }}^{⋆}\to \hat{\gamma }\hat{\omega }\right)$. Lasso regularization ensures sparsity in the deviations from the calibrated prior effects ($\sum_{j=1}^{p}I[{\hat{\beta }}_{j}\neq 0]\ll p$)—in contrast to ridge regularization—but not in the combined coefficients ($\sum_{j=1}^{p}I[\beta_{j}^{⋆}\neq 0]\;≲\;p$). As the combined coefficients may deviate more from unreliable than from reliable calibrated prior effects, this extension is suitable for partially informative co-data. As opposed to ‘standard stacking’, we refer to this extension as ‘simultaneous stacking’. Algorithm 1 includes the pseudo-code for both approaches.

Algorithm 1. Pseudo-code for the proposed transfer learning method with standard stacking (left) and simultaneous stacking (right). Sub-procedures (italicized) are explained below.

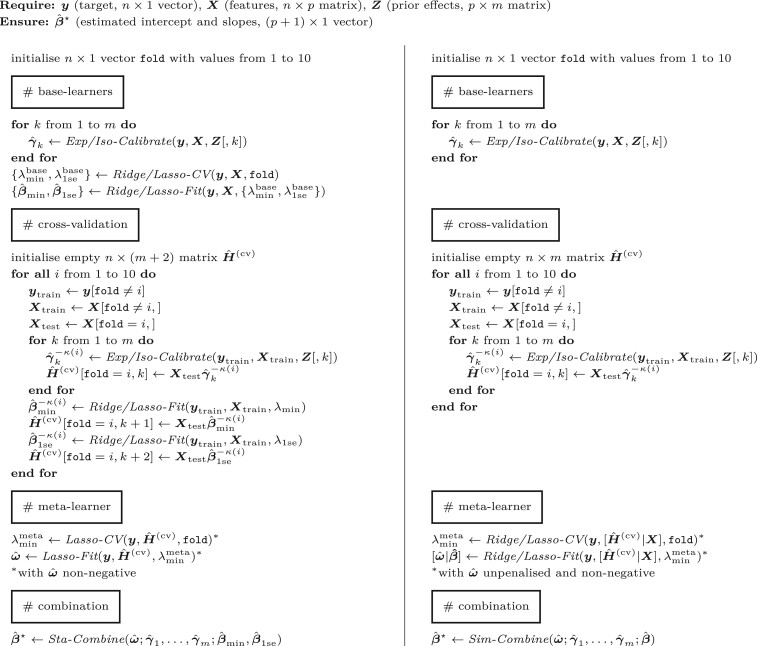

- *Exp/Iso-Calibrate* ${}^{†}$ : Performs exponential or isotonic calibration (Section). Requires target vector, feature matrix, and prior effects. Ensures calibrated prior effects.
- *Ridge/Lasso-CV* ${}^{†}$ : Tunes the hyperparameter of ridge or lasso regression by *k*-fold cross-validation. Requires target vector, feature matrix, and fold identifiers. Ensures optimal regularization parameter.
- *Ridge/Lasso-Fit* ${}^{†}$ : Estimates the parameters of ridge or lasso regression. Requires target vector, feature matrix, and regularization parameter. Ensures estimated coefficients.
- *Sta/Sim-Combine*: Combines estimated parameters from standard stacking (Equation 1) or simultaneous stacking (Equation 2). Requires estimated parameters from base-learners and meta-learner. Ensures combined estimates.

${}^{†}$ Note: The procedures *Exp/Iso-Calibrate*, *Ridge/Lasso-CV*, and *Ridge/Lasso-Fit* depend on the family of distributions (Gaussian versus binomial), but they also require choices (exponential versus isotonic, ridge versus lasso).

## 3 Simulation

We performed two simulation studies to compare the predictive performance between our transfer learning method and the one from Tian and Feng (2022). In contrast to the method from Tian and Feng (2022), which requires the feature-target effects in the target and the source dataset(s) to be *positively correlated* and on the *same scale* (i.e. $\beta_{\text{target}}\approx \beta_{\text{source}}$), our method also allows for negatively correlated effects and for effects on different scales (i.e. $\beta_{\text{target}}\approx c\times \beta_{\text{source}}$). Although it is possible to overcome this restriction by inverting the target (Gaussian: $y_{\text{source}}\to -y_{\text{source}}$, binomial: $y_{\text{source}}\to 1-y_{\text{source}}$), by re-scaling the target (Gaussian: $y_{\text{source}}\to 1/c\times y_{\text{source}}$), or by inverting or re-scaling the features ($X_{\text{source}}\to c\times X_{\text{source}}$), we believe it is more user-friendly to directly allow for negative correlations and different scales. To ensure a fair comparison between the two methods, we simulate positively correlated effects on the same scale. Furthermore, although the method from Tian and Feng (2022) is in theory also suitable for mixed response types, the current version of the related R package glmtrans requires the source dataset(s) and the target dataset to have the same response type (Gaussian, binomial, Poisson). We therefore always simulate the same response type in the source and target domains. In addition, we also compared our method with the one from Kawaguchi *et al.* (2022, xrnet).

### 3.1 External simulation

We use the simulation approach from Tian and Feng (2022). In each iteration, we call the function glmtrans::models with the arguments (1) family of distributions: family= ”gaussian” (default) or family=”binomial”, (2) source or target datasets: type=”all” (default), (3) difference between source and target coefficients: h = 5 (default) or h = 250, (4) number of source datasets: K = 5 (default), (5) sample size for target dataset: n.target = 100 (default), (6) sample size for each source dataset: n.source = 150 (default), (7) number of non-zero coefficients: s = 15 (default) or s = 50, (8) number of features: p=1000 (default), number of transferable source datasets: Ka = 1, Ka = 3 or Ka=K = 5 (default).

The simulation from Tian and Feng (2022) involves the following steps:

- Features: The correlation between features *i* and *j* is set to $\Sigma_{ij}=0.5^{\left|i-j\right|}$, where *i* and *j* are in {1,…,p}. Let Σ represent the correlation matrix and let $\Sigma =R^{⊺}R$ represent its Cholesky decomposition, where ***R*** is an upper triangular matrix. For the target dataset ($n_{0}=100$) and each source dataset ($n_{1}=\cdots =n_{5}=150$), the $n_{0}\times p$ matrix $X_{0}=E_{0}R$ and the $n_{k}\times p$ matrices $X_{k}=E_{k}R$ for *k* in {1,…,5} represent the features, where the $n_{0}\times p$ matrix $E_{0}$ and the $n_{k}\times p$ matrices $E_{k}$ contain Gaussian noise.
- Coefficients: Let *β_j_* represent the effect of feature *j*, for *j* in {1,…,p}, and denote the *p*-dimensional coefficient vectors by $\beta_{0}$ for the target dataset and $\left\{\beta_{1},\ldots ,\beta_{5}\right\}$ for the source datasets. For the *target* dataset, the first *s* elements are set to $\beta_{j}=0.5$ (causal) and the last *p–s* elements are set to $\beta_{j}=0$ (non-causal). For *transferable source* datasets, the first *s* elements are set to $\beta_{j}=0.5+{\left(-1\right)}^{z_{j}}h/p$ and the last *p–s* elements are set to $\beta_{j}={\left(-1\right)}^{z_{j}}h/p$, where *z_j_* is a realization of $z_{j}∼\text{Bernoulli}(0.5)$. For *non-transferable source* datasets, the first *s* elements are set to $\beta_{j}={\left(-1\right)}^{z_{j}}2h/p$ (non-causal), the next *s* elements are set to $\beta_{j}=0.5+{\left(-1\right)}^{z_{j}}2h/p$ (causal), and the last p−2s elements are a random sample of *s* causal and p−3s non-causal elements generated in the same way. To obtain a non-transferable source, it would be sufficient to randomly select causal elements rather than inverting causal and non-causal elements (indices 1 to 2*s*).
- Targets: In the *Gaussian* case, the target vector is the *n*-dimensional vector $y_{0}=X_{0}\beta_{0}+\epsilon_{0}$ for the target dataset, and the *n*-dimensional vector $y_{k}=0.5+X_{k}\beta_{k}+\epsilon_{k}$ for source dataset *k*, where the *n*-dimensional vectors $\left\{\epsilon_{0},\ldots ,\epsilon_{5}\right\}$ contain Gaussian noise. In the *binomial* case, let $p_{0}=1/(1+\text{exp}(-X_{0}\beta_{0}))$ for the target dataset and $p_{k}=1/(1+\text{exp}(-0.5-X_{k}\beta_{k}))$ for the source datasets. The *n*-dimensional vectors $\left\{y_{0},\ldots ,y_{5}\right\}$ are the target vectors, with each element following a Bernoulli distribution with the probability given by $\left\{p_{0},\ldots ,p_{5}\right\}$.

### 3.2 Internal simulation

The simulation from Tian and Feng (2022) uses the same effect size for all causal features and a decreasing correlation structure with a fixed base. We therefore designed our own data-generating mechanism (i) to simulate different effect sizes for different causal features ($\beta_{j}\in R$ instead of $\beta_{j}\in \{0,0.5\}$) and (ii) to vary the strength of correlation between features ($\sigma_{ij}=\rho_{x}{}^{\left|i-j\right|}$ instead of $\sigma_{ij}=0.5^{\left|i-j\right|}$).

Our simulation involves the following steps:

- Features: Setting the mean of feature *i* to $\mu_{i}=0$, the variance of feature *i* to $\sigma_{ii}=1$, and the covariance between features *i* and *j* to $\sigma_{ij}=\rho_{x}^{\left|i-j\right|}$, for all *i* and *j* in {1,…,p}, we simulate multiple feature matrices from the multivariate Gaussian distribution with mean vector μ and covariance matrix Σ, namely the $n_{0}\times p$ feature matrix $X_{0}$ for the target dataset, and the $n_{1/2/3}\times p$ feature matrices $\left\{X_{1},X_{2},X_{3}\right\}$ for the source datasets ($n_{0}=100,n_{1}=n_{2}=n_{3}=150,p=500$).
- Coefficients: Setting the mean and the variance for dataset *k* to $\mu_{k}=0$ and $\sigma_{kk}=1$, for all *k* in {0,1,2,3}, and the covariance between datasets *k* and *l* to $\sigma_{kl}=0$ if either *k* or *l* equals 1, or to $\sigma_{kl}=\rho_{\beta }$ if both *k* and *l* are in {0, 2, 3}, we simulate two p×4 matrices from the multivariate Gaussian distribution with mean vector μ and covariance matrix Σ, namely $B_{1}$ and $B_{2}$. We define the coefficients as $B=B_{1}I[B_{2}>\phi^{-1}(1-\pi )]$, where ϕ is the Gaussian cumulative distribution function and *π* equals 0.2 (dense) or 0.05 (sparse), and denote the *p*-dimensional coefficient vectors by $\beta_{0}$ for the target dataset and by $\left\{\beta_{1},\beta_{2},\beta_{3}\right\}$ for the source datasets. While one set of coefficients is non-transferable ($\beta_{1}$), we transform the transferable sets of coefficients with $\beta_{2}\to \text{sign}(\beta_{2})|\beta_{2}{|}^{2}$ and $\beta_{3}\to \text{sign}(\beta_{3})\sqrt{\left|\beta_{3}\right|}$.
- Targets: For the target dataset, we compute $z_{0}=X_{0}\beta_{0}$ and standardize $z_{0}$ to obtain $z_{0}^{*}$. For the source datasets, we proceed similarly to obtain $\left\{z_{1}^{*},z_{2}^{*},z_{3}^{*}\right\}$. The simulated targets equal $y_{k}=h^{-1}(\sqrt{w}z_{k}^{*}+\sqrt{1-w}\epsilon_{k})$, where h(·) is a link function and $\epsilon_{k}$ follows a standard Gaussian distribution, for *k* in {0,…,3}. Given 0≤w≤1, we have $\text{Var}(\sqrt{w}z_{k}^{*}+\sqrt{1-w}\epsilon_{k})=w\text{Var}(z_{k}^{*})+(1-w)\text{Var}(\epsilon_{k})=1$. While h(·) is the identity link in the Gaussian case, it is the logit link in the binomial case, where the simulated probabilities are rounded to simulated classes. We set the signal-to-noise ratio to 4:1 (*w* = 0.8).

### 3.3 Simulation results

In addition to the target dataset, the method from Tian and Feng (2022, glmtrans) requires the source datasets, while our method (transreg) and the method from Kawaguchi *et al.* (2022, xrnet) require the prior effects derived from the source datasets. Since these methods have different requirements, we first simulate the source datasets (for glmtrans) and then derive the prior effects from the simulated source datasets (for xrnet and transreg). As prior effects, we use the estimated coefficients from penalized regression on the source datasets. We choose the type of regularization for all methods subject to the simulation setting, namely ridge regularization for dense settings (glmtrans: *α* = 0; xrnet and transreg: $\alpha_{\text{source}}=\alpha_{\text{target}}=0$) and lasso or lasso-like elastic net regularization for sparse settings (glmtrans: *α* = 1; xrnet and transreg: $\alpha_{\text{source}}=0.95,\;\alpha_{\text{target}}=1$). The idea of the lasso-like elastic net regularization is to render the prior information more stable.

In each simulation setting, we simulate 100 training samples and 10 000 testing samples (hold-out) for the target dataset. Tables 3 and 4 show the predictive performance for the testing data in the external and internal simulation, under exponential and isotonic calibration. We observe that transfer learning (with glmtrans, xrnet, or transreg) often leads to a significant improvement with respect to standard penalized regression (with glmnet). Concerning the proposed method, this holds for the two different calibration approaches and the two different stacking approaches. These simulation studies do not show that the proposed transfer learning method outperforms other transfer learning methods. The advantage of the proposed method as compared to the method from Tian and Feng (2022, glmtrans) is that it does not require the source data but only the prior effects derived from the source data, and the advantage as compared to the method from Kawaguchi *et al.* (2022, xrnet) is that it allows for non-linear relationships between the prior effects and the true effects. While the method from Kawaguchi *et al.* (2022, xrnet) shrinks the coefficients towards a linear function of the prior effects, the proposed method adapts the prior effects to the true effects through exponential or isotonic calibration.

**Table 3.** Predictive performance in external simulation.^a^

									transreg			
*K*_a_	*h*	α	Family	${\bar{\rho }}_{x}$	$\max({\hat{\rho }}_{\beta })$	glmnet	glmtrans	xrnet	exp.sta	exp.sim	iso.sta	iso.sim
1	5	0	Gaussian	0.01	1.00	73.2 ± 3.0	43.9 ± 9.2*	47.1 ± 1.8*	32.2 ± 3.2*	30.9 ± 2.8*	24.5 ± 3.6*	23.4 ± 3.3*
3	5	0	Gaussian	0.01	1.00	73.2 ± 3.0	33.5 ± 6.5*	29.3 ± 2.4*	18.1 ± 2.5*	16.7 ± 2.0*	13.5 ± 2.0*	12.7 ± 1.6*
5	5	0	Gaussian	0.01	1.00	73.2 ± 3.0	24.0 ± 3.7*	22.6 ± 1.5*	14.2 ± 1.7*	13.2 ± 1.7*	10.6 ± 1.0*	10.0 ± 0.9*
1	250	0	Gaussian	0.01	0.40	73.2 ± 3.0	31.8 ± 9.4*	63.8 ± 3.6*	54.8 ± 6.0*	57.8 ± 6.5*	49.5 ± 6.9*	50.0 ± 7.3*
3	250	0	Gaussian	0.01	0.42	73.2 ± 3.0	33.3 ± 8.2*	51.4 ± 4.7*	46.5 ± 7.9*	47.0 ± 9.2*	39.4 ± 6.5*	37.6 ± 7.5*
5	250	0	Gaussian	0.01	0.43	73.2 ± 3.0	33.3 ± 5.0*	43.4 ± 3.4*	40.0 ± 6.3*	39.2 ± 7.3*	32.7 ± 4.4*	30.0 ± 4.7*
1	5	1	Gaussian	0.01	1.00	17.3 ± 3.8	12.5 ± 1.4*	14.6 ± 1.7	12.2 ± 1.9*	14.7 ± 1.9*	11.0 ± 1.6*	11.6 ± 1.6*
3	5	1	Gaussian	0.01	1.00	17.3 ± 3.8	10.5 ± 0.7*	11.5 ± 0.6*	10.8 ± 0.6*	11.7 ± 1.6*	9.8 ± 0.5*	9.9 ± 0.6*
5	5	1	Gaussian	0.01	1.00	17.3 ± 3.8	10.0 ± 0.4*	11.0 ± 0.6*	10.5 ± 0.5*	11.1 ± 1.3*	9.6 ± 0.3*	9.8 ± 0.4*
1	250	1	Gaussian	0.01	0.24	17.3 ± 3.8	22.2 ± 13.4†	17.6 ± 4.1	14.7 ± 3.4*	17.8 ± 4.8	14.7 ± 3.4*	18.2 ± 5.0
3	250	1	Gaussian	0.01	0.27	17.3 ± 3.8	19.8 ± 6.7†	17.7 ± 3.6†	14.7 ± 3.4*	18.4 ± 4.8	14.8 ± 3.4*	18.7 ± 5.5†
5	250	1	Gaussian	0.01	0.27	17.3 ± 3.8	19.8 ± 6.7†	17.5 ± 3.6	14.8 ± 3.3*	19.0 ± 4.6	14.7 ± 3.5*	21.3 ± 6.6†
1	5	0	Binomial	0.01	1.00	91.5 ± 1.5	91.0 ± 4.2	84.5 ± 1.0*	82.4 ± 5.6*	82.7 ± 5.4*	77.8 ± 3.7*	78.5 ± 4.7*
3	5	0	Binomial	0.01	1.00	91.5 ± 1.5	85.9 ± 3.7*	75.9 ± 2.9*	69.8 ± 3.6*	70.0 ± 3.8*	66.7 ± 3.7*	66.6 ± 4.5*
5	5	0	Binomial	0.01	1.00	91.5 ± 1.5	79.9 ± 2.4*	70.8 ± 2.9*	63.1 ± 3.8*	63.2 ± 3.9*	61.2 ± 3.0*	62.2 ± 5.7*
1	250	0	Binomial	0.01	0.41	91.5 ± 1.5	92.0 ± 5.0	91.1 ± 2.0	89.4 ± 2.9*	90.2 ± 3.0	88.3 ± 3.2*	88.7 ± 3.0*
3	250	0	Binomial	0.01	0.42	91.5 ± 1.5	89.7 ± 4.3	86.5 ± 4.2*	86.3 ± 4.6*	87.8 ± 4.5*	84.0 ± 7.5*	84.2 ± 5.6*
5	250	0	Binomial	0.01	0.43	91.5 ± 1.5	85.3 ± 3.5*	83.0 ± 3.2*	84.3 ± 4.8*	85.9 ± 5.5*	79.4 ± 4.4*	79.0 ± 5.0*
1	5	1	Binomial	0.01	1.00	80.4 ± 8.2	77.8 ± 12.2	77.1 ± 8.4	71.4 ± 5.8*	73.0 ± 4.0*	70.3 ± 5.2*	72.4 ± 5.0*
3	5	1	Binomial	0.01	1.00	80.4 ± 8.2	70.5 ± 12.5*	69.5 ± 11.8*	67.9 ± 5.7*	70.8 ± 10.3*	64.7 ± 4.8*	64.4 ± 5.1*
5	5	1	Binomial	0.01	1.00	80.4 ± 8.2	59.5 ± 2.5*	64.4 ± 4.2*	65.7 ± 6.4*	65.5 ± 9.6*	62.7 ± 4.5*	61.8 ± 4.2*
1	250	1	Binomial	0.01	0.25	80.4 ± 8.2	82.1 ± 8.7†	82.4 ± 9.9†	81.5 ± 9.2	81.5 ± 9.1	81.2 ± 7.2	82.4 ± 10.1†
3	250	1	Binomial	0.01	0.26	80.4 ± 8.2	80.5 ± 9.3	82.0 ± 8.8†	80.7 ± 8.8	81.3 ± 9.5	79.2 ± 8.3	82.7 ± 11.2
5	250	1	Binomial	0.01	0.27	80.4 ± 8.2	78.9 ± 9.4*	84.6 ± 15.5	80.3 ± 8.3	83.4 ± 8.8†	79.4 ± 10.7	86.3 ± 16.8†

a In each setting (row), we simulate 10 datasets, calculate the performance metric (mean-squared error for numerical prediction, logistic deviance for binary classification) for the test sets, express these metrics as percentages of those from prediction by the mean, and show the mean and standard deviation of these percentages. Settings: number of transferable source datasets (*K_a_*), differences between source and target coefficients (*h*), dense setting with ridge regularization (*s *=* *50, *α* = 0) or sparse setting with lasso regularization (*s *=* *15, *α* = 1), family of distribution (‘gaussian’ or ‘binomial’). These parameters determine (i) the mean Pearson correlation among the features in the target dataset (${\bar{\rho }}_{x}$) and (ii) the maximum Pearson correlation between the coefficients in the target dataset and the coefficients in the source datasets ($\max({\hat{\rho }}_{\beta })$). Methods: regularized regression (glmnet), competing transfer learning methods (glmtrans, xrnet), proposed transfer learning method (transreg) with exponential/isotonic calibration and standard/simultaneous stacking. In each setting, the colour black (grey) highlights methods that are more (less) predictive than regularized regression without transfer learning (glmnet), asterisks (daggers) indicate methods that are *significantly* more (less) predictive at the 5% level (one-sided Wilcoxon signed-rank test), and an underline highlights the most predictive method.

**Table 4.** Predictive performance in internal simulation.^a^

									transreg			
*ρ_x_*	$\rho_{\beta }$	*α*	Family	${\bar{\rho }}_{x}$	$\max({\hat{\rho }}_{\beta })$	glmnet	glmtrans	xrnet	exp.sta	exp.sim	iso.sta	iso.sim
0.95	0.60	0	Gaussian	0.08	0.32	46.1 ± 7.3	48.5 ± 6.3	45.2 ± 7.0*	45.2 ± 7.6*	45.4 ± 7.1	45.1 ± 7.5*	44.5 ± 6.8*
0.99	0.60	0	Gaussian	0.32	0.32	33.2 ± 4.8	33.2 ± 6.4	32.6 ± 4.4*	32.2 ± 4.6*	32.5 ± 4.4*	32.1 ± 4.7*	32.0 ± 4.1*
0.95	0.80	0	Gaussian	0.08	0.54	45.1 ± 6.7	47.9 ± 11.1	42.0 ± 6.4*	43.7 ± 7.1*	43.3 ± 6.9*	43.8 ± 7.1*	41.9 ± 6.5*
0.99	0.80	0	Gaussian	0.32	0.54	32.5 ± 4.8	31.8 ± 4.2	31.1 ± 4.7*	32.0 ± 4.8*	31.6 ± 4.7*	31.6 ± 4.7*	30.4 ± 3.9*
0.95	0.99	0	Gaussian	0.08	0.89	44.1 ± 5.1	42.2 ± 5.6	37.3 ± 4.4*	40.9 ± 5.3*	37.6 ± 4.5*	40.1 ± 5.7*	38.8 ± 5.7*
0.99	0.99	0	Gaussian	0.32	0.89	31.0 ± 5.1	29.4 ± 2.1	27.6 ± 3.0*	30.4 ± 5.3	28.4 ± 3.8*	29.8 ± 4.8*	29.1 ± 3.9*
0.95	0.60	1	Gaussian	0.08	0.27	37.7 ± 6.8	39.1 ± 7.2	37.9 ± 6.6	37.3 ± 7.2	38.0 ± 7.3	37.4 ± 7.0	41.5 ± 6.4†
0.99	0.60	1	Gaussian	0.32	0.27	28.5 ± 2.4	29.9 ± 3.5†	29.1 ± 3.4	28.8 ± 2.5	29.0 ± 2.7	28.7 ± 2.7	28.7 ± 2.5
0.95	0.80	1	Gaussian	0.08	0.45	37.5 ± 4.8	37.4 ± 5.0	36.9 ± 5.2	35.7 ± 4.2*	36.8 ± 4.7	35.9 ± 4.7*	39.5 ± 5.4†
0.99	0.80	1	Gaussian	0.32	0.45	29.9 ± 2.4	29.9 ± 2.8	29.5 ± 3.4	29.1 ± 2.5	29.4 ± 3.3	29.1 ± 2.8	29.6 ± 3.8
0.95	0.99	1	Gaussian	0.08	0.87	38.0 ± 6.4	33.0 ± 5.7*	34.5 ± 7.9*	34.8 ± 8.2*	34.1 ± 6.8*	34.0 ± 7.6*	35.2 ± 8.0
0.99	0.99	1	Gaussian	0.32	0.87	30.2 ± 4.4	29.5 ± 4.5	29.2 ± 4.6	29.4 ± 4.8	29.1 ± 4.2*	28.6 ± 3.5*	29.4 ± 4.4
0.95	0.60	0	Binomial	0.08	0.32	77.1 ± 4.5	81.2 ± 6.1†	76.7 ± 4.4	76.8 ± 4.8	76.1 ± 5.0	77.7 ± 4.8	77.7 ± 5.2
0.99	0.60	0	Binomial	0.32	0.32	65.5 ± 4.7	67.0 ± 5.9	65.0 ± 4.7	63.7 ± 4.8*	65.3 ± 4.4	63.8 ± 4.8*	64.0 ± 4.9
0.95	0.80	0	Binomial	0.08	0.54	74.9 ± 4.4	81.2 ± 10.7†	73.5 ± 5.1*	75.0 ± 5.7	73.9 ± 5.6	74.7 ± 4.7	73.5 ± 5.4*
0.99	0.80	0	Binomial	0.32	0.54	64.3 ± 3.6	64.7 ± 4.6	61.8 ± 4.7*	63.2 ± 4.5*	63.7 ± 5.0	63.0 ± 4.7*	63.6 ± 5.1
0.95	0.99	0	Binomial	0.08	0.89	75.4 ± 5.0	74.8 ± 4.7*	69.5 ± 4.2*	72.4 ± 6.5*	71.5 ± 6.3*	71.7 ± 4.9*	71.4 ± 4.8*
0.99	0.99	0	Binomial	0.32	0.89	62.4 ± 5.0	61.8 ± 5.0	58.1 ± 4.6*	61.0 ± 6.7	58.9 ± 6.7*	60.4 ± 6.2*	59.6 ± 5.0*
0.95	0.60	1	Binomial	0.08	0.27	76.5 ± 6.0	75.8 ± 3.9	75.7 ± 4.1	75.8 ± 5.8	76.5 ± 4.9	75.9 ± 5.4	75.9 ± 2.3
0.99	0.60	1	Binomial	0.32	0.27	61.2 ± 4.4	61.5 ± 4.9	63.4 ± 6.8†	63.1 ± 5.6†	62.2 ± 5.7	62.7 ± 5.4†	61.5 ± 4.6
0.95	0.80	1	Binomial	0.08	0.45	78.2 ± 10.5	76.6 ± 10.2	76.3 ± 9.8	75.0 ± 6.0	77.1 ± 9.0	75.2 ± 6.1	77.9 ± 6.6
0.99	0.80	1	Binomial	0.32	0.45	64.9 ± 4.9	64.8 ± 5.6	65.5 ± 2.9	66.3 ± 6.7	64.4 ± 5.2	65.2 ± 6.0	65.5 ± 4.3
0.95	0.99	1	Binomial	0.08	0.87	80.1 ± 6.0	73.3 ± 5.7*	70.7 ± 6.3*	69.2 ± 4.5*	68.8 ± 6.8*	70.1 ± 5.2*	69.3 ± 5.3*
0.99	0.99	1	Binomial	0.32	0.87	63.2 ± 5.1	62.9 ± 4.7	61.2 ± 5.6*	62.7 ± 6.1	61.1 ± 5.7*	61.8 ± 5.8	61.2 ± 6.2*

a In each setting (row), we simulate 10 datasets, calculate the performance metric (mean-squared error for numerical prediction, logistic deviance for binary classification) for the test sets, express these metrics as percentages of those from prediction by the mean, and show the mean and standard deviation of these percentages. Settings: correlation parameter for features (*ρ_x_*), correlation parameter for coefficients ($\rho_{\beta }$), dense setting with ridge regularization (π=30%, *α* = 0) or sparse setting with lasso regularization (π=5%, *α* = 1), family of distribution (‘gaussian’ or ‘binomial’). These parameters determine (i) the mean Pearson correlation among the features in the target dataset (${\bar{\rho }}_{x}$) and (ii) the maximum Pearson correlation between the coefficients in the target dataset and the coefficients in the source datasets ($\max({\hat{\rho }}_{\beta })$). Methods: regularized regression (glmnet), competing transfer learning methods (glmtrans, xrnet), proposed transfer learning method (transreg) with exponential/isotonic calibration and standard/simultaneous stacking. In each setting, the colour black (grey) highlights methods that are more (less) predictive than regularized regression without transfer learning (glmnet), asterisks (daggers) indicate methods that are *significantly* more (less) predictive at the 5% level (one-sided Wilcoxon signed-rank test), and an underline highlights the most predictive method.

## 4 Applications

### 4.1 External applications

First, we consider an adapted version of the application on cervical cancer from van de Wiel *et al.* (2016). The aim is to transfer information from a methylation study with biopsy samples to another methylation study with self-collected samples in order to better discriminate between low-grade and high-grade precursor lesions. Specifically, we transfer the signs of the effect sizes and the *P*-values from the source dataset to the target dataset (*n *=* *44 samples, p=9491 features). We then examine whether this prior information increases the predictive performance of ridge regression, which is more predictive than lasso regression in this application. Next to our transfer learning method (transreg) and the one from Kawaguchi *et al.* (2022, xrnet), we consider the co-data learning methods from Tay *et al.* (2023, fwelnet) and van Nee *et al.* (2021, ecpc). While these transfer learning methods exploit information on the importance and direction of the effects (co-data: $-\text{sign}(\text{coef}){\;\text{log}\;}_{10}(P-\text{value})$), these co-data learning methods only exploit information on their importance (co-data: $-{\;\text{log}\;}_{10}(P-\text{value})$). After 10 repetitions of 10-fold cross-validation, we observe that the proposed method (not with exponential but with isotonic calibration) often increases the predictive performance of ridge regression (transreg.exp.sta: 0/10, transreg.exp.sim: 4/10, transreg.iso.sta: 7/10, transreg.iso.sim: 10/10, fwelnet: 7/10, ecpc: 5/10, xrnet: 3/10). We also observe that exploiting information on the importance as well as the direction of the effects (transreg) can be more beneficial than exploiting information on the importance of the effects only (fwelnet, ecpc), as can be seen in the mean change in cross-validated logistic deviance (transreg.exp.sta: +6.56%, transreg.exp.sim: +0.43%, transreg.iso.sta: −2.50%, transreg.iso.sim: −9.25%, fwelnet: −0.12%, ecpc: −1.52%, xrnet: +2.59%). The relatively poor performance of the competing transfer learning method (xrnet) might be caused by a non-linear relationship between the prior effects (transformed *P*-values) and the true effects. Here, isotonic calibration outperforms exponential calibration, and simultaneous stacking outperforms standard stacking. A potential explanation for the large difference in performance between exponential and isotonic calibration is that positive effects might be more important than negative effects in this application, for a biological reason (methylation increases the probability of cancer) and a statistical reason (effects of overexpression are easier to detect than those of underexpression). While exponential calibration behaves symmetrically for negative and positive prior effects, isotonic calibration can shrink negative prior effects towards zero.

Second, we consider an adapted version of the application on pre-eclampsia from Tay *et al.* (2023). Measurements of p=1125 plasma proteins are available for *n *=* *166 patients at multiple time points (48×2+8×3+20×4+74×5+16×6=666). The aim is to transfer information from late time points (gestational age > 20 weeks) to early time points (gestational age ≤20 weeks). We repeatedly split the patients into one source dataset and one target dataset. Patients with only late time points are always in the source dataset, and other patients are randomly allocated to the source and the target dataset. (Note that this application is somewhat artificial, as it might be better to drop transfer learning in favour of using all earliest time points in the regression of interest.) Using the source dataset, we estimate two logistic regression models under ridge regularization, once using the early time points and once using all time points. For each patient, all time points are assigned to the same cross-validation fold, and the weight is split evenly among the time points. We then use the two sets of estimated regression coefficients as co-data for the target dataset. In the regression for the target dataset, we only include the earliest time point of each patient. Using 10-fold cross-validation, we estimate the predictive performance of ridge regression with and without transfer learning. After repeating source-target splitting and cross-validation 10 times, we observe that transfer learning tends to decrease the cross-validated logistic deviance (transreg.exp.sta: 8/10, transreg.exp.sim: 7/10, transreg.iso.sta: 7/10, transreg.iso.sim: 9/10, fwelnet: 5/10, ecpc: 6/10, xrnet: 10/10). It is more beneficial to share information not only on the importance but also the direction of the effects, according to the mean change in cross-validated logistic deviance (transreg.exp.sta: −2.61%, transreg.exp.sim: −4.29%, transreg.iso.sta: −3.67%, transreg.iso.sim: −8.33%, fwelnet: +0.04%, ecpc: −6.87%, xrnet: −12.12%). Simultaneous stacking again outperforms standard stacking, but exponential and isotonic calibration show a similar performance. In this application, where the prior effects are probably close to the true effects, the competing transfer learning method (xrnet) outperforms the proposed one.

**Figure 2.**
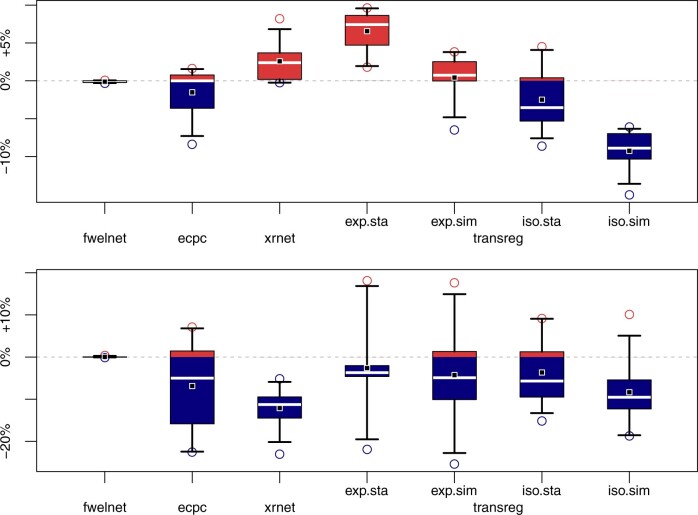
Change in predictive performance in external applications due to prior information. Percentage change in cross-validated logistic deviance (*y*-axis) from ridge regression (with glmnet) to other methods (*x*-axis), for 10 repetitions of 10-fold cross-validation. A negative value (blue) indicates an improvement, and a positive value (red) indicates a deterioration, based on the metric ’logistic deviance’. Top: application on cervical cancer. Bottom: application on pre-eclampsia. From left to right: co-data learning methods (fwelnet, ecpc), competing transfer learning method (xrnet), proposed transfer learning method (transreg) with exponential/isotonic calibration and standard/simultaneous stacking.

For both applications, Fig. 2 shows the change in predictive performance from modelling without to modelling with prior information.

### 4.2 Internal application

In this application, we transfer information from a meta-analysis of genome-wide association studies on Parkinson’s disease (PD-GWAS, Nalls *et al.* 2019) to the Luxembourg Parkinson’s study (LuxPARK, Hipp *et al.* 2018). The aim is to classify samples into Parkinson’s disease (PD) patients and healthy controls based on SNPs.

At the time of our study, the LuxPARK dataset included genotyping and clinical data of 790 PD cases and 766 healthy controls. DNA samples were genotyped using the NeuroChip array (Blauwendraat *et al.* 2017). Quality control steps of genotyping data were conducted according to the standard procedures reported previously (Pavelka *et al.* 2022). Missing genotyping data were imputed using the reference panel from the Haplotype Reference Consortium (release 1.1) on the Michigan Imputation Server (Das *et al.* 2016) (RRID: ID_017579), with a filter for imputation quality (*r*^2^ > 0.3).

As common SNPs exhibit weak effects on PD, the sample size is likely insufficient to train a highly predictive model. However, publicly available summary statistics from the largest-to-date PD-GWAS (with around 38 000 cases and 1 400 000 controls from European ancestry) (Nalls *et al.* 2019) might serve as prior information on the SNP effects. For each SNP, these summary statistics are the combined results from simple logistic regression of the PD status on the SNP, namely the estimated slope (logarithmic odds ratio), its standard error, and the associated *P*-value. Importantly, the LuxPARK cohort was not part of the PD-GWAS, meaning that the prior information comes from independent data. As the LuxPARK cohort and the PD-GWAS cohorts have a similar ethnic background, the prior information might allow us to increase the predictive performance.

The two lists of SNPs—from the LuxPARK genotyping data (target dataset) and the PD-GWAS summary statistics (source dataset)—are partially overlapping. SNP data are high-dimensional and strongly correlated. From each block of SNPs in the target dataset (250 kb window), we retain the most significant one and those that are in weak pairwise linkage disequilibrium with it ($r^{2}<0.1$). Next, we only retain the SNPs appearing also in the source dataset. These two filtering steps together reduce the dimensionality in the target dataset from around 18 million SNPs to 196 018 SNPs. We code the SNP data for dominant effects, with 0 meaning no alternate allele (0/0) and 1 meaning one or two alternate alleles (0/1 or 1/1).

It seems that the results from the source dataset are informative, because 5.80% of the *P*-values are nominally significant at the 0.05 level (11 377 out of 196 018), 77 are significant at a false discovery rate of 5% (Benjamini–Hochberg), and 35 are significant at a family-wise error rate of 5% (Holm–Bonferroni). As SNPs with a low minor allele frequency might have large effect sizes but insignificant *P*-values, we base the prior effects not on the estimated coefficients ($\hat{\beta }$) but on the signed logarithmic *P*-values ($-\text{sign}(\hat{\beta }){\;\text{log}\;}_{10}(P)$). For each SNP, we compared the reference and the alternate alleles between the two datasets: (i) if both datasets have the same reference allele and the same alternate allele, the signed logarithmic *P*-value from the source dataset becomes the prior effect for the target dataset; (ii) if the reference allele of each dataset is the alternate allele of the other dataset (swapped alleles), we invert the sign of the signed logarithmic *P*-value; and (iii) if the two datasets have two different sets of alleles (multiallelic SNP), we set the prior effect to zero.

Rather than using the 196 018 SNPs for predictive modelling in the target dataset, we also filter them based on their significance in the source dataset (which is already a type of transfer learning). For each cut-off in $\left\{5\times 10^{-2},5\times 10^{-3},\ldots ,5\times 10^{-10}\right\}$, we exclude all SNPs above and include all SNPs below. This means that for the target dataset, we retain a specific number of the most significant SNPs from the source dataset. For each significance cut-off, we compare three modelling approaches:

- *Uninformed approach*: We use logistic regression with ridge or lasso penalization to model the PD status based on the included SNPs. All included SNPs are treated equally, irrespective of their estimated effect in the source dataset.
- *Naïve transfer learning*: After calculating for each sample the sum across the signed logarithmic *P*-values from the source dataset multiplied by the SNPs from the target dataset, we fit a simple logistic regression of the PD status on this sum.
- *Transfer learning*: The proposed transfer learning approach uses the signed logarithmic *P*-values from the source dataset as prior effects for the target dataset.

**Figure 3.**
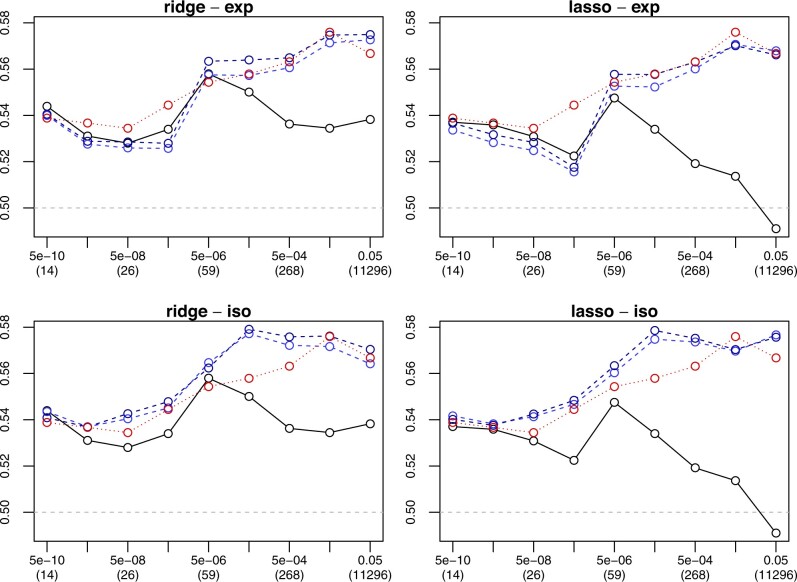
Predictive performance in internal application. Mean cross-validated area under the ROC curve (AUC) from 10 times 10-fold cross-validation (*y*-axis) against *P*-value cutoff (*x*-axis) for regression without (solid line) and with (dashed lines) transfer learning (bright blue: standard stacking, dark blue: simultaneous stacking), under either ridge (left) or lasso (right) regularization and either exponential (top) or isotonic (bottom) calibration. The numbers within brackets indicate the dimensionality, and the dotted line is for naïve transfer learning. While AUC=0.5 represents random classification, AUC=1.0 represents perfect classification. Given 766 controls and 790 cases, a random classifier achieves an AUC above 0.524 in 5% of the cases.

Figure 3 shows the predictive performance of modelling with estimated effects (uninformed approach), with prior effects (naïve transfer learning), or with both (transfer learning). We obtained the results with repeated nested cross-validation (10 repetitions, 10 external folds, 10 internal folds), using the same folds for all methods. If the significance cut-off is very strict, leading to a small number of significant SNPs, transfer learning does not improve the predictive performance of ridge and lasso regression. In these low-dimensional settings with many fewer SNPs than samples, prior information on the SNPs is not helpful. But otherwise transfer learning does improve the predictive performance of ridge and lasso regression. This holds for all four flavours of the proposed transfer learning method (exponential versus isotonic calibration, standard versus simultaneous stacking), but isotonic calibration works considerably better than exponential calibration and simultaneous stacking works marginally better than standard stacking.

Depending on the significance cut-off determining the number of significant SNPs, the performance of naïve transfer learning can be as high as the one of transfer learning with isotonic calibration. In these cases, the prior effects are predictive to the extent that it is not even necessary to estimate any effects. An explanation for the high performance of naïve transfer learning might be (i) the large sample size in the source dataset for testing the marginal effects of the SNPs together with (ii) the linkage disequilibrium clumping leading to a selection of relatively independent SNPs.

## 5 Discussion

We proposed a two-step transfer learning method for exploiting estimated coefficients from related studies to improve the predictive performance in the study of interest. First, we adapt the prior effects from the source datasets to the target dataset, either with exponential or isotonic calibration. While exponential calibration is more robust to outliers (only three free parameters), isotonic calibration is more flexible (only maintains order of prior effects). We expect the former to be superior if the prior effects are close to the true effects, and the latter to be superior if there is no exponential relationship. Second, we combine the calibrated prior effects with information from the observed data, based on two variants of stacked generalization. While the first variant (standard stacking) is more suitable if there are many sources of co-data (‘averaging calibrated prior effects and estimated effects’), the second variant (simultaneous stacking) is more suitable if there is one source of co-data with partially unreliable or partially missing prior effects (‘shrinking combined effects towards calibrated prior effects’).

The proposed transfer learning method allows for multiple sources of prior information. It does not require the source dataset(s) but only the prior effects derived from the source dataset(s). The proposed sequential transfer learning method has a competitive predictive performance with the less flexible parallel transfer learning method from Tian and Feng (2022, glmtrans). In the case of closely related tasks, accounting for prior effects with transfer learning seems to be more beneficial than accounting for prior weights with the co-data methods from Tay *et al.* (2023, fwelnet) and van Nee *et al.* (2021, ecpc). And in the case of non-linearly related prior effects, the proposed method seems to outperform the one from Kawaguchi *et al.* (2022, xrnet). We therefore believe that the proposed method could tackle many biomedical predictions problems with one or more sets of prior effects.

In some applications, only one type of prior information derived from the source datasets is available. In other applications, multiple types of prior information are available (or the source datasets themselves). Then we can choose from multiple types of prior information. If the source and target datasets have the same feature space, estimated coefficients from penalized regression might be a reasonable choice. If the feature spaces are different, however, it is problematic that (i) lasso regression erratically selects among correlated features and (ii) ridge regression distributes weight among correlated features. This means that the presence or absence of additional correlated features in the source datasets might change the prior information on the features of interest. The same problem arises under contamination of a subset of features (van de Wiel *et al.* 2016). We therefore expect that signed logarithmic *P*-values from pairwise testing ($-\text{sign}(\hat{\beta }){\;\text{log}\;}_{10}(P)$) will often be more informative than estimated coefficients from multiple regression ($\hat{\beta }$).

## Data Availability

The R package transreg is available on GitHub (https://github.com/lcsb-bds/transreg) and CRAN (https://cran.r-project.org/package=transreg), with the code for the simulations and the applications in a vignette (https://lcsb-bds.github.io/transreg/). We obtained our results using R 4.3.0 (RRID: ID_001905) on a physical machine (aarch64-apple-darwin20, macOS Ventura 13.5.2). Data for the application on cervical cancer are available from van de Wiel *et al.* (2016), in the R package GRridge in the dataset ‘dataVerlaat’ (source data: Farkas *et al.* 2013, target data: van de Wiel *et al.* 2016). Data for the application on pre-eclampsia are available from Erez *et al.* (2017), in the supporting file ‘pone.0181468.s001.csv’. For the application on Parkinson’s disease, the source data are available from Nalls *et al.* (2019), in the online file ‘nallsEtAl2019_excluding23andMe_allVariants.tab’, and the target data are available upon request (request.ncer-pd@uni.lu). Information on reproducibility is also available on a frozen page (doi: 10.17881/hczj-3297).
